# IoT Sensing Platform as a Driver for Digital Farming in Rural Africa

**DOI:** 10.3390/s20123511

**Published:** 2020-06-21

**Authors:** Antonio Oliveira-Jr, Carlos Resende, André Pereira, Pedro Madureira, João Gonçalves, Ruben Moutinho, Filipe Soares, Waldir Moreira

**Affiliations:** 1 Fraunhofer Portugal AICOS, 4200-135 Porto, Portugal or antoniojr@ufg.br (A.O.-J.); carlos.resende@fraunhofer.pt (C.R.); andre.pereira@fraunhofer.pt (A.P.); pedro.madureira@fraunhofer.pt (P.M.); joao.goncalves@fraunhofer.pt (J.G.); ruben.moutinho@fraunhofer.pt (R.M.); filipe.soares@fraunhofer.pt (F.S.); 2 Institute of Informatics (INF)—Federal University of Goiás (UFG), Goiânia 74690-900, Brazil

**Keywords:** sensing platform, computer vision, small-scale farmers, internet of things, digital farming, rural Africa

## Abstract

Small-scale farming can benefit from the usage of information and communication technology (ICT) to improve crop and soil management and increase yield. However, in order to introduce digital farming in rural areas, related ICT solutions must be viable, seamless and easy to use, since most farmers are not acquainted with technology. With that in mind, this paper proposes an Internet of Things (IoT) sensing platform that provides information on the state of the soil and surrounding environment in terms of pH, moisture, texture, colour, air temperature, and light. This platform is coupled with computer vision to further analyze and understand soil characteristics. Moreover, the platform hardware is housed in a specifically designed robust casing to allow easy assembly, transport, and protection from the deployment environment. To achieve requirements of usability and reproducibility, the architecture of the IoT sensing platform is based on low-cost, off-the-shelf hardware and software modularity, following a do-it-yourself approach and supporting further extension. In-lab validations of the platform were carried out to finetune its components, showing the platform’s potential for application in rural areas by introducing digital farming to small-scale farmers, and help them delivering better produce and increasing income.

## 1. Introduction

It is evident how agriculture can benefit from the usage of information and communication technology (ICT) with farming-related processes, in the whole value chain, being optimized (e.g., from seeding to reaching the shelves), resulting in better produce and increased yields, a much desired outcome to cope with the ever-increasing population and food demand. ICT’s relevance in the agriculture domain is reflected in the investments and granted projects on the topic of rural digital transformation (2014-2020 EU contribution 192M€, 436 participation in selected projects [1] as well as in the expected market value of digital farming solutions (USD 10+bn by 2025) [2].

Despite of the important role of small-scale farming in worldwide food production [3,4], family farmers see very little of the aforementioned investments and projects focusing on their reality, added to the fact that small-scale farmers are in disadvantage from many perspectives—lack of proper tools, non-compliance with large-scale farming standards, low income, difficult access to inputs (e.g., fertilizer, seeds, nutrients), no access to technological tools, to name a few.

Upon this, we propose an Internet of Things (IoT) sensing platform that is a collection of software, and low-cost sensors and wireless communication, to make digital farming accessible to small-scale farmers in rural Africa [5]. This platform can be broken down into three main components, namely (i) the sensing box that follows a Do-It-Yourself (DIY) approach which is based on off-the-shelf hardware and extensible software; (ii) the computer vision that comprises the software for controlling the camera and illumination system to capture soil images, and for classifying the soil type; and (iii) the protective casing that shelters all the hardware, allowing easy assembly, usage and transportation of platform.

The proposed IoT sensing platform is part of the Project AFRICA [6] that aims at developing a green-energy driven technology solution to support the on-site, cost-affordable fertiliser production to small-scale farmers in Africa. Thus, reducing the yield gap these farmers experience due to the difficult access (i.e., their low income, high prices) to fertilizers.

The main contributions of this paper can be summarized as follows:

- Sensing box based on low-cost, off-the-shelf hardware and software modularity, following a DIY approach for further extension without requirements of extended hardware and software development knowledge.
- Soil classifier based on computer vision with soil images acquired by a dedicated camera and controlled light system.
- Standardized 3D-printed protective casing to safeguard all hardware components from the extreme environment conditions, and focused on easy production, assembly and simple integration of the proposed solution.
- In-lab validation of each component of IoT sensing platform, namely the sensing box, the computer vision, and the casing, targeting further finetuning and improvements to allow a single, integrated platform robust enough for the target application scenarios.

This paper is structured as follows—Section 2 presents the background information on how ICTs enable digital farming and overviews the digital farming initiatives that could be employed in Africa. Then, Section 3 provides detailed information on the proposed IoT sensing platform and its components, namely sensing box, computer vision and protective casing. Next, Section 4 presents the initial and final prototypes of the sensing platform, going over the performed set of validations for improving the proposed solution. Finally, Section 5 concludes the paper.

## 2. Background and Related Work

This section provides a general view of ICT enabling digital farming, illustrating its different applications. Then, it focus directly to a list of IoT platforms, implementations and deployments targeting digital farming with potential application in Africa, and that are somewhat related to the IoT sensing platform we propose in this paper.

### 2.1. How ICT Enables Digital Farming

Digital farming is an ever-expanding field focused on the enhancement of agriculture through improved information and communication processes. This involves conceptualizing, designing, developing, evaluating and applying innovative ways to use ICTs in the agriculture domain. ICTs help enhancing agriculture productivity by manifolds. The popularization of wireless technology, sensors, computer vision and image analysis, positioning systems, machine learning (ML), artificial intelligence (AI), among others play a significant role in delivering digital farming to monitor and manage agriculture processes.

Within the context of agriculture, the role of digital farming is twofold. First, it shall provide the means for the farmer to measure, as accurately and cost-effective as possible, relevant variables, such as the weather, soil characteristics (e.g., pH, moisture, and nutrient content), external factors (e.g., competition to his crops from weeds, threats to their health from pests and diseases). Then, it must produce relevant information to help the farmer take informed decisions regarding his fields, crops, and livestock (e.g., when and how much to irrigate, safe application of pesticides, suitable amounts of fertilizers, when to feed and administer medicines).

Upon this, this section provides a general background on how ICTs have been enabling digital farming in a variety of use cases and application scenarios. It is worth to mention that the goal of this section is not to provide an exhaustive analysis of digital farming solutions, but rather help building the background knowledge towards what is applying ICT in farming. For a comprehensive survey we point the reader to Reference [7].

Farmnote^®^ (Japanese startup), GAMAYA^®^ (spin-off from École Polytechnique Fédérale de Lausanne (EPFL)) and Green Sense Farms Holdings^®^ are examples of solutions that rely on gathering sensor data, image and data analysis, and AI to keep track of livestock, to improve farming processes in a more efficient and sustainable way, and to automate farming practices.

For solutions considering the usage of the cloud, robots and sensors with the goal of providing a single source of data, controlling robots, and automating processes in dairy farms, one can may come across systems such as MyAgData^®^, FarmBot^®^, and Mastiline LUCI^®^. Solutions such as TerrAvion^®^, Semios^®^, and Blue River Technology combine image analysis, cloud solutions, sensing, data and predictive analytics, computer vision and AI in order to improve revenue based on precision agriculture, keep track of plant health and manage pest control, and provide a smart machine system that take take decisions at plant level.

With special attention to pest control and real-time monitoring of crops, Pycno Agriculture Sensors^®^, Resson^®^, and Prospera^®^ provide solutions aiming at improved productivity and that are based on IoT sensors, data and predictive analytics, computer vision, ML, image and data analysis.

Regarding Decision Support Systems (DSS) powered with ML, predictive analytics, AI, statistical analysis, and drone and satellite imagery, one may come upon PowWow^®^, aWhere^®^, Ec2ce^®^, and FluroSat^®^ that provide DSS solutions to monitor different farming-related perspective (e.g., plants, crops, weather, supply chain, market) in order to allow informed decision regarding the farming processes, guide investments, and improve crop performance. Sencrop^®^, Descartes Labs^®^, and CowLar Smart^®^ focus either on the state of the fields or of livestock. These solutions bring together sensor and cloud infrastructure, AI, and data analysis to understand the current state of plants, animals, and weather, also providing forecasts and recommendations to allow for taking informed decisions.

Be it for indoor and outdoor or for soil and hydroponic-based farming, Grownetics^®^, Hortal^®^, Agrivi^®^, and Alesca Life^®^ provide solutions looking at different farming processes (e.g., planting, irrigation, harvesting). The solutions rely on sensors data analysis, and cloud infrastructure to help manage and optimize the target crops.

Finally, Saturas^®^, GreenSeeker handheld crop sensor^®^, and FieldScout GreenIndex+ Nitrogen App and Board^®^ rely on DSSs, sensors, and smartphone-based applications to gather data on the plants and soil. The data provides insights into the health and needs of the plants and soil, offering the means for taking informed decisions to improve irrigation and fertilization, and to yield better quality produce. Generally speaking, the aforementioned ICT-based solutions look at farming and respective processes from different perspectives as presented next.

- Type of farming: whether the solutions target rearing of animals or crop cultivation;
- Purpose of applied technology: focusing on the main goal of the employed solution in the farming processes, that is, monitoring, actuation, control, and automation;
- Expected outcomes: aiming at better yields, pest and disease control, quality standards, safe transportation, improved storage, nutrient levels, health status, sustainable process.
- Considered technology: referring to the employed ICT concepts to derive the solutions, that is, sensing, communication networks, computer vision, cloud infrastructure, mobile applications, AI, ML, unmanned vehicles and robots, and so on.

This shows how the multifaceted features of ICT enable digital farming. It also serves our purpose of not only having a broader view of digital farming, but also identifying where the proposed IoT sensing platform fits. That is, a solution targeting crop cultivation based on sensor and communication networks, and computer vision to monitor soil conditions to help small-scale farmers achieve better yields. With this background on digital farming at hand, next we provide an overview of different IoT platforms, implementations and deployments targeting e-Agriculture for Africa.

### 2.2. Digital Farming for Africa

General IoT adoption in Africa has been discussed [8,9,10] and one can observe that, despite the low penetration of IoT in Africa, there are different efforts to encourage the adoption of IoT-related technologies. For instance, Gichamba et al. [11] propose the use of a mobile application to assess the impact that m-Agriculture solutions have among the small-scale farmers in Kenya. Sousa et al. [12] and Oliveira et al. [13] implement a communication infrastructure and a set of community tools, respectively. The ultimate goal of both works is the introduction of ICT to improve the quality of life of users in rural areas in Mozambique.

Moreover, we can identify different works that implement digital farming that are or could be applied in Africa, and that are related to the proposed IoT sensing platform. However, for a more detailed overview of IoT-based solutions and platforms, we again direct the reader to Reference [7].

Combining sensors, long-range communication, cloud infrastructure, and a mobile application, the WAZIUP project [14] delivers an open platform for water, soil, and waste management in Western Africa countries (i.e., Ghana, Senegal, and Togo). Analogous to the proposed IoT sensing platform, WAZIUP follows a DIY approach to reduce costs of the solution. The main difference resides in the fact that our solution was meant to be portable, not relying on a gateway (i.e., the smartphone is the data sink, and later synchronizes with the backend server as further explained in Section 3).

FarmBeats [15] focuses on data gathering from sources such as sensors, cameras, and drones. The propose IoT-based solution monitors soil and facilities (i.e., storage, shelters) to improve yields, minimize losses, and reduce costs. While the solution is a fully integrated IoT platform, associated costs may not be affordable for small-scale farmers as the proposed IoT sensing platform.

The IoTecture [16] is an architecture focusing on five layers, namely device, transport, data, model and service. The proposed architecture also takes into account the location where components are deployed, that is, at the thing, mist, fog, cloud, and terminal. As one can observe, IoTecture combines different ICT approaches, with the goal of allowing the deployment of smart applications in different domains, including agriculture. The proposed IoT platform differs from IoTecture as it targets a much less complex scenario, but could be seen as a component of the latter.

Also focusing on water management, the SWAMP project [17] brings together communication technologies, sensing, data acquisition protocols and software, data bases, optimization models, and application services. The goal is to provide a solution that is easily adapted, customized, and replicable according to different scenarios’ needs. Analogous to the IoT sensing platform, SWAMP targets replication and adaptability, but they differ in the fact that the former goes beyond water management, focusing on providing the means for suitable fertilization of soil for improved yields.

Another way to closely monitor crop conditions and help providing data for informed decisions is to consider Wireless Sensor Networks (WSNs). Haseeb et al. [18] propose a IoT-based WSN framework, with a special focus on how data is transmitted and an associated level of security. Secured data transmission is an important topic, and the proposed IoT sensing platform reduces security issues by considering data transmission only to one sink at the time (i.e., the smartphone).

On the same line of combining IoT with WSNs, Garcia et al. [19] provide a detailed overview on how IoT-based WSNs can be employed to deliver irrigation in a more sustainable way. The overview covers relevant parameters concerning irrigation (e.g., water, soil, and weather characteristics), commonly used technologies, and challenges and practices associated to irrigation solutions. The proposed IoT platform can be seen as way of providing means for sustainable water utilization, as it allows us to understand the needs of the soil, and can be used to trigger irrigation actuators solely when necessary.

Also proposing a low-cost solution, Abba et al. [20] proposes a system based on Arduino, sensors, and irrigation-related hardware to monitor water needs of the soil and control irrigation actuators. While the proposed solution is very close to our work, the proposed IoT sensing platform differs in as it is meant to sense soil needs to not only support a sustainable usage of water, but also to help in the production and application of fertilizers.

By combining low-cost sensors, drones, and data fusion, Spachos [21] proposes a system to monitor soil in order to identify conditions that could lead to the appearance of pests and disease in viticulture. Similar to our solution, the system relies on sensor data and image analysis. However, the difference relates to the fact that the image analysis carried out by the proposed IoT sensing platform targets the provision of more information on the soil.

From the aforementioned digital farming-based solutions and that have potential application in Africa, one can see that they resort to IoT-based platforms, sensor networks and solutions from different perspectives and a varied set of goals. This shows the multipurpose features of IoT-related solutions when applied in the digital farming domain.

However, none of the initiatives have been focused on providing an integrated IoT sensing platform that is easy to reproduce, deploy and use by users, with no to little digital literacy and low income, to seamlessly obtain soil data in rural Africa.

## 3. Proposed IoT Sensing Platform

As aforementioned, the proposed IoT sensing platform is comprised of—(i) the sensing box (a collection of sensors and the respective software and the host hardware); (ii) the computer vision module (machine learning algorithms on soil images captured, and the best classification model to deploy), and the protective casing (that turns the platform into a single, robust box).

The sensing box is composed of sensors that provide numeric readings on specific soil and environment parameters (i.e., pH, moisture, air temperature, light) to help triggering the fertiliser production, and to keep farmers/soil experts aware of the soil current status. The computer vision component resorts to camera and illumination control for capturing images and analyzing them with algorithms to provide information on about the soil type where the IoT sensing platform is used. Given the extreme environments on which the platform is expected to be employed (e.g., high temperatures, heavy rainfall) and the fact that is built based on low-cost, sensitive hardware, the platform resorts to its protective casing component to shield the inner parts of the solution.

This section starts by presenting the architecture of the IoT sensing platform, followed by a detailed overview of each component.

### 3.1. Architecture

**Figure 1:**
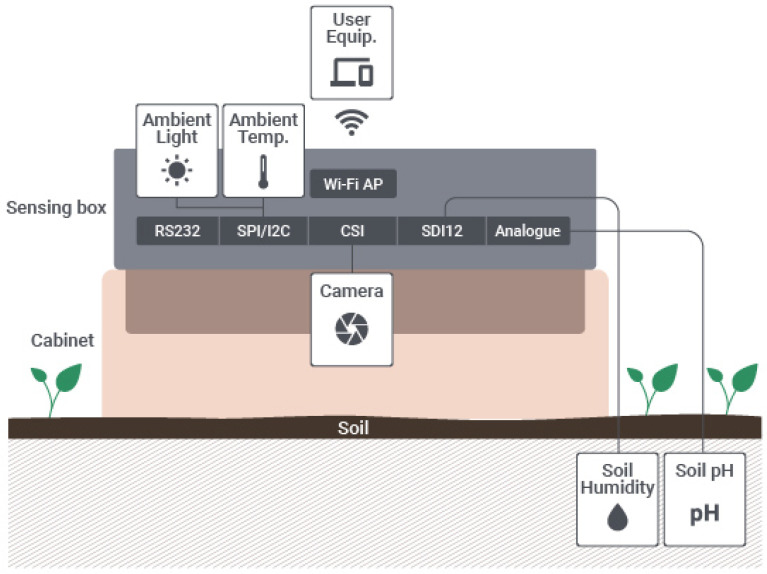
Internet of Things (IoT) sensing platform high-level architecture.

Figure 1 presents the high-level architecture of the IoT sensing platform with its collection of sensors and interfaces, as well as the radio communication interface, which allows the collected data to be relayed to a user equipment (UE) (e.g., smartphone, tablet, laptop), according to the specificities of application use cases. Moreover, the architecture showcases the camera and the cabinet that isolates from natural light the soil to be captured, and later analysed.

As illustrated in Figure 1, the architecture considers that the IoT sensing platform solely exchange data with a UE, and this is done exclusively through the Wi-Fi interface (the sensing box functions as a Wi-Fi Access Point to which the UE connects). It is the user equipment that serves as relay to deliver the sensed data to the backend server. This architecture is further detailed considering its hardware and software perspectives in Section 3.2.

**Figure 2:**
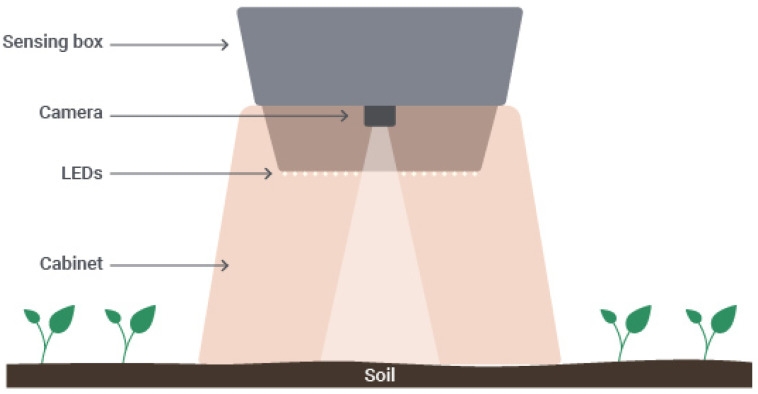
Structure for the soil image acquisition.

In what concerns the computer vision component, Figure 2 illustrates the structure that relies on the camera sensor, LEDs and cabinet for soil image acquisition, further detailed in Section 3.3.

Finally, the design details of the 3D-printed protective casing proposed to house all the hardware (i.e., single board computer (SBC), microcontroller, sensors, illumination, mounting parts) that compose the IoT sensing platform are presented in Section 3.4.

### 3.2. Sensing Box Component

The sensing box component was designed with technology acceptance and reproducibility features in mind. As for the former feature, the sensing box is built based on set of sensors to which soil specialists are used in their daily routine. While the latter feature relates to the fact that the box can be easily reproduced in each target country, considering locally found hardware and avoiding cumbersome importation and customs processes.

This section starts by introducing the set of sensors and probes to be considered for building the sensing box, followed by its final hardware and software architectures.

#### 3.2.1. Analysed Sensors and Probes

The main requirements for considering the following sensors and probes (to be incorporated or connected into the sensing box) were that they be: (i) familiar to soil specialists; (ii) low-cost; (iii) not complex to integrate in the sensing box; and iv) easily found in the local hardware market.

It is worth noting that the information presented in this section comes from the input provided by the National Agriculture Research Organization (NARO) in Uganda, and the Nelson Mandela University (NMU) in South Africa. Both NARO and NMU are partners in Project AFRICA, and are located in the target countries for deployment of the proposed IoT sensing platform. NARO has shared two brands of sensing products normally used by their soil experts:

- LaMotte [22]—offers a set of soil testing kits and instrumentation equipment. The testing kits are reagent-based products, offering a visual colour matching system to monitor the soil status. This is a disposable solution only possible to integrate in the sensing box by an automatic analysis of the colour matching system. The instrumentation equipment is composed by a unique system that incorporates the sensing probe and a display to show the measured values. They do not provide any communication interface, so its integration in the sensing box is not possible due to associated complexity.
- pH Sensor by Mettler Toledo [23]—offers laboratory grade pH sensor. Some of the pH probes—as for example the pH electrode InLab Solids [24] and pH electrode InLab Solids Go-ISM [25]—are designed to be used in semi solid material, so they could be used to access obtain the pH status of the soil. They provide an analogue interface over multiple physical interfaces, so they can be integrated in the sensing box due to simplicity.

In addition to manufacturers shared by NARO, it was possible to identify *Jumia^®^*, an online retailer present in the Ugandan market that sells sensors for DIY projects, which can be used as a source of low-cost hardware to integrate in the sensing box. As for NMU, the shared information can be divided into three groups:

- DIY sensors—low-cost sensors with low precision and robustness levels. They can be used in budget-constrained implementations, where the sensor precision and robustness are not mandatory requirements;
- Commercial grade sensors—sensors sold for commercial applications. More expensive than the DIY sensors, but more reliable and robust;
- Soil analysis kits—reagent-based products that offer a visual colour matching system to monitor the soil status.

Considering the information provided, the strategy followed for the development of the sensing box was to evaluate the sensors shared by both NMU and NARO. Thus, we considered the sensors that could be seamlessly integrated in the sensing box and easily used by soil experts, while at the same time being low-cost and available locally. When the sensors failed to comply with the aforementioned requirements, we identified alternatives in the local distributors, comparing them with the ones shared by NARO and NMU and considering those that guarantee compliance and increase the acceptance of the technology by the soil experts.

Next, we present the sensing box hardware architecture that resulted from this close analysis on the sensors and probes provided by our Ugandan and South African partners.

#### 3.2.2. Detailed Overview of the Sensing Box Hardware

After understanding what sensors could be used to build the sensing box, we followed with the definition of the hardware architecture to guide its integration development. The rationale that guided such definition was the idea to allow, virtually, any soil expert (or other user with no electronics knowledge) to locally buy the necessary components and connect them in order to build the sensing box on their own. Considering this, we decided to follow a DIY approach for the solution, which ended up being composed by components that can be easily plugged together without the need for complex hardware-related tasks.

**Figure 3:**
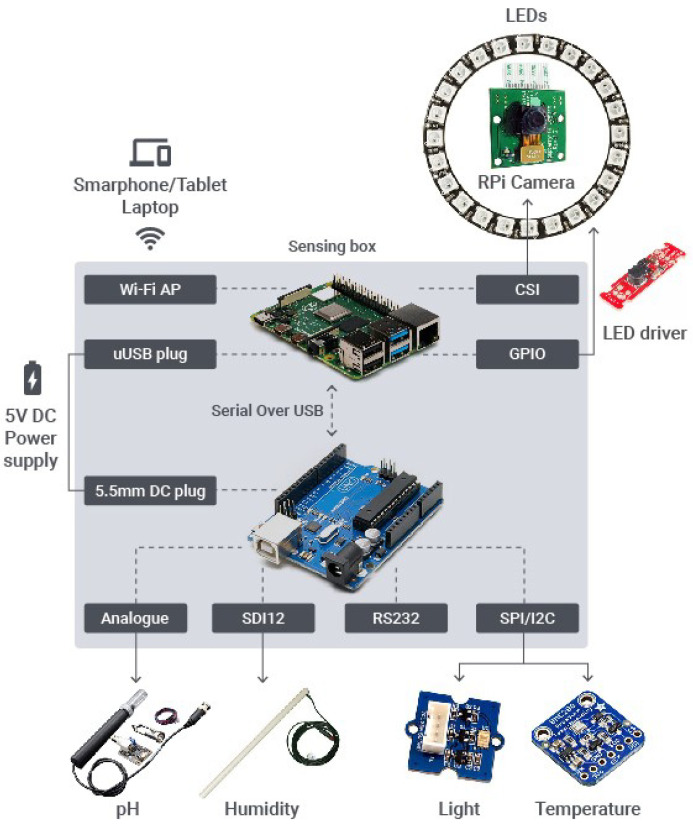
Sensing box hardware architecture.

The hardware architecture (cf., Figure 3) of the sensing box can be broken down into three main components as follows. A Raspberry Pi^®^ as the brain of the sensing box. This is a low-cost SBC commonly used in DIY projects. This device already comes with built-in Wi-Fi support that can be configured in access point (AP) mode, and can easily interface with the camera module. The 8 mega pixel camera module selected (Pi NoIR Camera V2) [26] is capable of 1080p video and still images and connects directly to Raspberry Pi through CSI (Camera Serial Interface) port on Raspberry Pi using a ribbon cable. This SBC is the element responsible for interfacing with the UE by providing a Wi-Fi AP to which such devices can connect to, as well as to interface with the camera through CSI and signalling the LED driver activation through the GPIO interface in the image acquisition process. The arrangement and number of LEDs are estimated using the beam angle or field-of-view (FOV) of a single LED. This measures how the light is distributed from the source of illumination onto a target area at certain distance. Using FOV in combination with the distance and target area we need to illuminate, the correct number of LEDs can be chosen. The beam angle for the selected LEDs (C513A-WSN-CY0Z0231) is 55º, at a distance of 330 mm from the target. The maximum target area is the same size of a A4 paper which theoretically gives a minimum of four LEDs to have a close to uniform illumination. It is worth noting that the arrangement and number of LEDs presented in Figure 3 are solely for illustration purposes. They differ from the proof of concept and final prototype, as illustrated in Section 4.1.4 and Section 4.2, following the identified needs to improve the illumination system while the work progressed.

An Arduino^®^ that interfaces with all soil and environment sensors. This is done through a header with screw connectors and protoboard space [27], where the SDI12, SPI/I2C, RS232 and analogue interfaces are provided. The reason to use Arduino for this task instead of the Raspberry Pi, is because the latter does not support SDI12 protocol [28], while Arduino does, since it allows a low-level control of the microcontroller, its timers and interrupts. Additionally, updating the Arduino software modules is much easier than updating the software modules on the Raspberry Pi. This further helps us achieving the desired DIY feature, which allows users with basic knowledge in software and hardware to update the sensors drivers, or even change the default sensors supported by the sensing box. Arduino is also a platform often used in DIY projects, so it is easily found in the hardware market. Its interface with Raspberry Pi is done using a standard USB cable, since both devices support serial communication on their USB ports.

A 5V DC power supply, which can be composed of standard AC/DC power adaptors compatible with Raspberry Pi and Arduino, or a standard power bank with power specification compatible with Raspberry Pi and Arduino power requirements (Raspberry Pi: Operating voltage 5V with 2.5A to 3A; Arduino: recommended input voltage 7–12 V, but operating voltage of 5V).

All these components can be easily connected to each other using standard equipment and connection interfaces. In addition to the inner hardware of the sensing box, it was also defined the set of sensors to measure pH, moisture, air temperature, light:

- DFRobot SEN0249 analogue spear tip pH sensor [29]. This is a pH sensor for semisolid material with an analogue interface. It is connected to the Arduino through the screw connectors on the Arduino header.
- Sentek drill & drop soil moisture, salinity and temperature probe [30]. This is a multi-depth industrial grade soil monitoring probe that communicates through SDI. It is connected to the sensing box through the SDI interface present in the screw connectors of the Arduino header. If cost or market availability may be an issue, a low-cost alternative to measure the soil moisture and temperature is also being explored with Seeed Studio 314010012 moisture and temperature sensor [31]. This sensor is connected to the sensing box through I2C interface present in the screw connectors of the Arduino header.
- Adafruit 2652 BME280 I2C/SPI temperature sensor [32]. It is worth mentioning that the Adafruit BME280 also provides humidity, barometric pressure, and altitude. This sensor is connected to the sensing box by soldering its pins into the protoboard space of the Arduino header.
- Seeed Studio (TSL2561) 101020030 digital light sensor [33]. This light sensor follows a DIY approach. It is connected to the sensing box through the I2C interface available in the screw connectors of the Arduino header.

The selection of this hardware to build the sensing box was performed having in mind a DIY approach, and aligned with the box’s requirements of familiarity, low-cost, simplicity and market availability on the target deployment countries.

#### 3.2.3. Detailed Overview of the Sensing Box Software

The same rationale used for the definition of the sensing box hardware was applied for the definition of its software architecture. The objective was to provide a sensing box that is capable of collecting soil and environment parameters, and that can also be easily adapted to new application requirements by users with low to medium level of hardware and software development knowledge. On the hardware side, this was accomplished following a DIY approach, and on the software side we achieve this by providing a set of software modules, and predefined communication interfaces and protocols, that can be easily adapted and extended according the new application scenarios and use cases.

**Figure 4:**
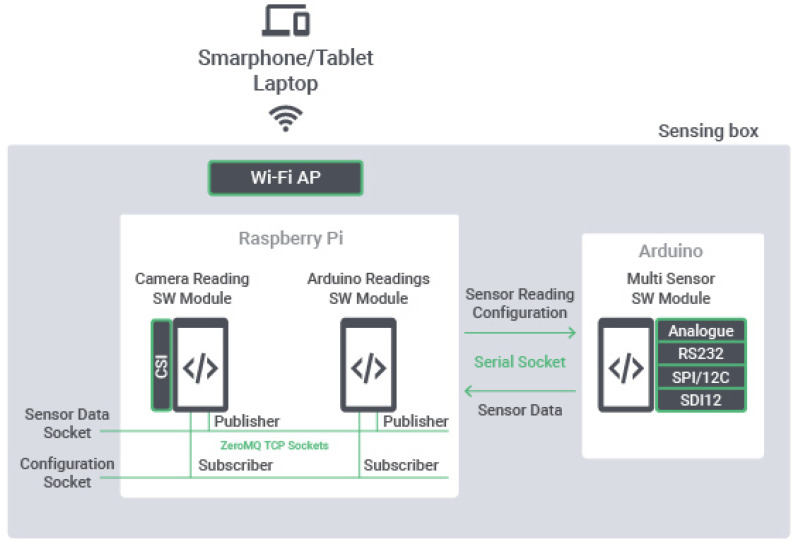
Sensing box software architecture.

Figure 4 presents the developed architecture, which is composed of the three software modules and three communication sockets. The interface with the UE is achieved by using the Raspberry Pi to provide a Wi-Fi access point where these devices connect to, and once connected they communicate with the software modules via the available ZeroMQ TCP sockets [34].

The sensing box software architecture is composed of three software modules, with two running in the Raspberry Pi (Camera Reading SW Module and Arduino Readings SW Module) and one running in the Arduino (Multi Sensor SW Module). These SW modules communicate between each other via three sockets, with two for the communications between the Raspberry and the UE connected to it through the provided Wi-Fi AP (ZeroMQ Sensor Data and Configuration sockets), and one for the communication between the Raspberry Pi and the Arduino (Serial Socket).

The **ZeroMQ Sockets** available in the Raspberry Pi are composed of two types of sockets:

- A **Configuration Socket** dedicated to communicating configurations for the software modules. In this socket, it is published the configuration for each sensor (e.g., period and duration of readings). The software modules running on Raspberry Pi subscribe to this socket to receive this information and configure themselves according to the configuration received.
- A **Sensor Data Socket** dedicated to communicating the data acquired from the sensors. The software modules running on the Raspberry Pi publish in this socket the readings they receive from the sensors.
- The communication on these sockets follows a publish-subscribe paradigm, where the software running on the UE opens the Configuration Socket in publisher mode and publishes the configuration message, while the software modules in the Raspberry Pi open it in subscriber mode and consumes the configuration message.

The **Serial Socket** is the socket that establishes the communication between the Raspberry Pi and the Arduino, more precisely between the **Arduino Readings SW Module** and the **Multi Sensor SW Module**. In this socket, Sensor Reading Configurations are sent by the Arduino Readings SW Module to the Multi Sensor SW Module, while Sensor Data (i.e., the readings from the sensors) are sent by the Multi Sensor SW Module to the Arduino Readings SW Module.

The **Camera Reading SW Module** running on the Raspberry Pi is the module responsible for interfacing with the camera through the CSI interface. This module performs the image acquisition by controlling the camera and illumination, performs the classification of the soil by computer vision and publishes the numerical result of soil class (related with texture and colour) in the Sensor Data Socket, according to the configuration received in the Configuration Socket.

The **Arduino Readings SW Module** running on the Raspberry Pi is responsible to interface with Arduino through the Serial Socket. It configures Arduino according to the configurations received in the Configuration Socket, gets the sensor readings from the Arduino and publishes them in the Data Socket.

The **Multi Sensor SW Module** running on the Arduino is responsible for interfacing with the Sensing Box ambient and soil sensors, through the available physical interfaces (i.e., Analogue, RS232, SPI/I2C and SDI12). It implements the communication drivers for the sensors and gets the sensor readings according to the configurations received on the Serial Socket. The readings are then provided to the Arduino Readings SW Module through the Serial Socket.

The presented software architecture is ready to be easily integrated in the sensing box hardware and, straightforwardly, communicate with its default sensors, while providing a communication interface for the UE. In addition to this, the software architecture allows for an easy integration of new sensors just by including the sensor driver on the Arduino Multi Sensor SW Module, and its configuration and sensing data in the configuration and sensor data messages. With this, the addition of new sensors in the sensing box is limited to modifications on Arduino software, which is by design thought for low to medium level technical users.

Looking at the system as a whole, modifications are also needed in the software that runs on the UE and is responsible for interfacing with the sensing box. The UE needs to send to the sensing box the correct reading configurations (e.g., period and duration of readings, etc.) for the new sensor, and correctly interpret the returned readings. As the UE software is outside of the sensing box development scope, it is assumed that such developments are also thought for low to medium level technical users and can be easily adapted to new application scenarios and use cases.

### 3.3. Computer Vision Component

The computer vision component of the IoT sensing platform can be divided into the acquisition of soil images and the corresponding soil classification using artificial intelligence.

It is worth noting that the component was also thought to be compatible with the referred principles of technology acceptance and high reproducibility of hardware and software. The camera itself is a sensor that works in combination with a set of LEDs actuators that compose the illumination system (including the LED driver). The compatible camera module selected is also available in the African market at a low cost. The software responsible for controlling the hardware parts is running in the SBC and do not require any further configurations in the field.

#### 3.3.1. Algorithms and Datasets

Image texture refers to patterns by contrast variations and an inherent inhomogeneity in natural surfaces resulting from properties such as roughness, depth, illumination and colour.

General texture analysis can be used for feature extraction and later for classification. Edge detectors are common image processing methods to identify points in the image which brightness changes sharply and find discontinuities. Edge detectors [35] can be employed to produce image derivatives to allow measuring soil particle sizes. To determine them, statistical metrics from the resulting contours of edge detector are calculated, such as area, length of contours and their distribution.

The soil particles distribution is used for soil classification and thus serves as the estimator for water holding capacity and drainage, aeration, susceptibility to erosion and cation exchange capacity, pH buffering capacity. Alternatively, using the library Texture-Color-Geometry Feature Extraction (TCGFE) [36,37,38] developed by Fraunhofer Portugal AICOS, a total of 152 features can be extracted for each region of interest in the image, which can later be used for machine learning purposes. Alternatively, texture can also be indirectly measured by fractal dimension [39]. This set of techniques require a strong knowledge in how the image is acquired and the software calibration process can be hard for the target technical user.

On the other hand, Deep Learning [40] approaches have recently had a transformative impact in image analysis specifically learning complex patterns of interest for applications such as object recognition and scene understanding. Due to the large variability of soil characteristics in the world, we raised the hypothesis of applying machine learning with Convolutional Neural Networks to the context of soil images, acquired in the controlled conditions of our IoT sensing platform.

#### 3.3.2. Soil classification using Convolutional Neural Networks

Soil surveys are typically focused on observing and measure soil attributes and properties that were relatively stable or static in time, as the particle size distribution of soil, topsoil thickness or organic carbon matter. The particle size and spatial distribution of soil (soil texture) are properties which influence and control how water infiltrates, how nutrients, chemicals and dissolved substances adhere to surfaces and are retained or transformed, and how energy and matter enter into the soil and is stored or transmitted through it. Soil Texture is the fundamental physical and mechanical property of soils. Soil classification system is essential for the identification of soil properties. Expert systems can be very powerful tools in identifying soils quickly and accurately [41,42]. This type of manual approach requires a lot of time, therefore a fast and reliable automated system is needed.

**Figure 5:**
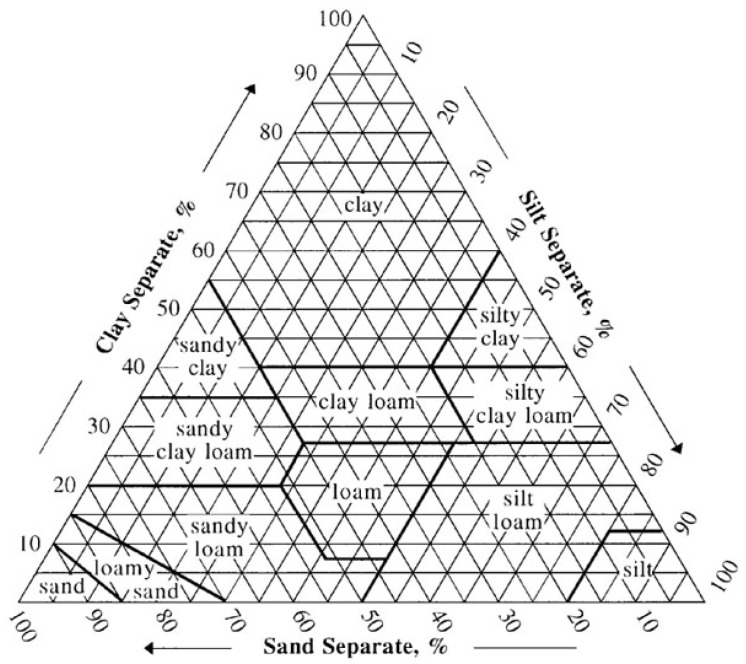
USDA soil texture triangle. A diagram with soil types according to their clay, silt and sand composition [45].

We propose an automated system that has been developed to classify soils in seven classes according to the Food and Agriculture Organization (FAO) World Reference Base soil groups (WRB), and USDA Soil Taxonomy suborders [43] presented in Figure 5. Our predictions are based on global spatial prediction models which we fitted, per topsoil images, using a compilation of major international soil profile databases. The dataset used was created from soil profiles from the World Soil Information Service (WoSIS) [44], which contains standardized and harmonized soil profile data and a range of global assessments. The images in this database are taken from soil profiles with a depth that varies between 0.8 m and 1.2 m.

Since we propose an automated system to classify topsoil properties, the images in the referred database are cropped in the topsoil part (about 30 cm in depth) in small images 300 × 300 pixels. The dataset created contains 18,493 images with multiple levels of categorical detail: the first level having 32 Reference Soil Groups (RSGs); the second level with 7 classes simplified according to the WRB; and the third level with 3 classes that are related with the percentage of sand, clay and silt. With this dataset various deep learning architectures are trained to classify in 3 classes (sand, clay or slit) and in 7 WRB classes for reference soil groups [43], which are:

1. Soils with strong human influence;
2. Soils with limitations to root growth;
3. Soils distinguished by Fe/Al chemistry;
4. Pronounced accumulation of organic matter in the mineral topsoil;
5. Accumulation of moderately soluble salts or non-saline substances;
6. Soils with clay-enriched subsoil;
7. Soils with little or no profile differentiation.

There are other public databases, such as Land Use and Cover Area frame Statistical (LUCAS) [46] over the extent of European Union (EU) and African soil information service (AfSIS) [47] aimed at collecting harmonized data about the state of land use or coverage, that can be considered to validate the algorithms produced in Project AFRICA. These services provide data and information to validate the computer vision models for image texture detection and classification. Later in the project, it is expected that the African partners produce specific datasets for the target deployment sites. In the current computer study we have used the top quality dataset found by WoSIS.

The deep learning architectures used pre-trained in imagenet images, are the VGG16 [48], InceptionV3 [49], MobileNetV2 [40] and NasNetMobile [50]. The architecture of our ColorTexture architecture was created for the proposed task consists 2 base networks with 4 blocks of 3 convolution layers then this group of filters is split in 2 get the maximum and minimum and then added again following by relu normalization [51] and max pooling for each block. The difference between the 2 base networks is the convolution process in one of the networks is a separable convolution process.

The models were trained using Adam optimizer [52], learning rate of 3 $\times 10{}^{-5}$ and clip norm of 0.001. In the training process, 11648 images are used 8154 for train and 3494 in the validation. Before the data is processed for training, it is resized to 224 × 224@3 and subtracted by mean image of the training data. In training process, the data is randomly mirrored to increase the number of images. We use a batch size of 16 and trained for 100 epochs. We use keras framework with tensorflow [53] backend to implement and train our models.

**Figure 6:**
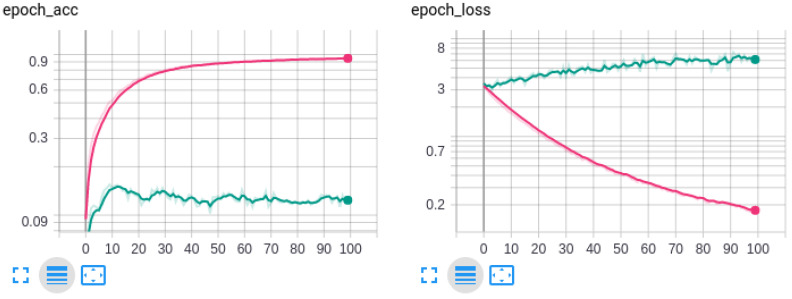
MobileNetV2 ACC and Loss (yy axis in log scale) along the epochs (xx axis) for train (red) and validation (green) for the 7 classes task

For testing purposes, we use about 37% of the dataset samples that are not used in the training process. Figure 6 shows that the train process had a larger degree of overfitting because the variance in the soils images is very low between distinct classes. We tried to prevent this overfitting with a spatial dropout layer that performs randomly “dropping out” (i.e., set to zero the activation for that neuron) with probability of 24%. This layer is added after the model last layers and before the classification layers. Even so, the overfitting is still present and we believe it can be due to the low spatial variability in the soils images between distinct classes. Table 1 and Table 2 present the summarized results of the experiments for validation and test datasets with five different architectures.

A first global conclusion that we can derive from the comparison of the Table 1 and Table 2 is that the performances achieved on both datasets is practically the same for the seven classes classification, the classification rate actually remains very low, with a maximum of 32.45% accuracy on the validation set and 28.25% accuracy on the test set. On the other side the classification task for 3 class-level the validation set archive better results than the test set. This may be a result of the intra-class variation that after the train does not generalize well in the test set.

Regarding the inference throughput, the computer vision component was also validated concerning image acquisition processing time and quality of the captured image. On the Raspberry Pi the loading time for model needs up to 20 second until the model is ready for inference, and the inference time needs about 5 second per image. Thus, in total the time needed to take a picture, preprocess and classify it is about 30 seconds. In this first experimentation, the model used on the Raspberry Pi has the MobileNetV2 deep learning architecture. We expect to optimize this time by converting the final best model with TensorFlow lite to efficiently execute the model on the Raspberry Pi.

### 3.4. Casing Component

In order to cope with the outdoor operation and possible deployments in extreme environments (e.g., high temperatures, heavy rainfall), the IoT sensing platform required a robust and protective casing to satisfactorily protect the hardware.

To ensure that the casing met the protection needs of the IoT sensing platform, we identified the main requirements that drove the design of the initial concept, namely: (i) prevent moisture and dust from reaching the hardware; (ii) robust outdoor use; (iii) compact; (iv) easy assembly (i.e., few elements, large parts, simple mounting); (v) based on locally available resources; (vi) easy usability; (vii) safe transport; viii) simple maintenance.

**Figure 7:**
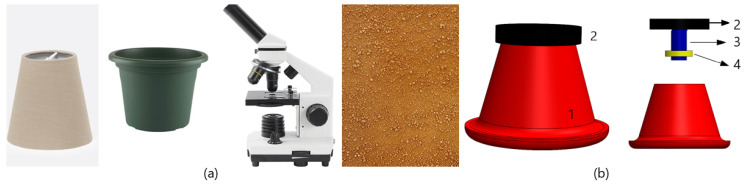
(**a**) Inspiration board. (**b**) Initial casing concept diagram: (1) Cabinet; (2) Sensing box casing; (3) Camera; (4) LEDs.

The design process started with the definition of an inspiration panel (cf., Figure 7a), focusing on objects that are useful in everyday life and able to satisfy the aforementioned requirements: a lampshade that absorbs light and creates light controlled environments. A vase with standard dimensions. The microscope object detail, used to obtain information through the visualization of samples. The control of distance, light, correct position, and data interpretation is an object of reference, that is, the soil.

With these requirements in mind and considering the inspiration board, we repurposed the utilization of the vase (hereafter referred to as cabinet), bringing a new meaning to it and aligning with requirement of locally found resources (i.e., a vase can be easily found anywhere). Within the context of the IoT sensing platform, this repurposed element not only serves to hold the sensing box, but also provides the controlled light zone for image acquisition performed by the computer vision component. Figure 7b illustrates this initial concept.

This initial concept considers the requirements of protection, simplicity and usability of the case that securely shelters the proposed IoT sensing platform, and that can be easily replicated anywhere in the world. Hence, the concept repurposes the use of a vase, turning it into a cabinet and giving it the function of a lampshade for blocking exterior light (requirement for image acquisition process for the computer vision component).

**Figure 8:**
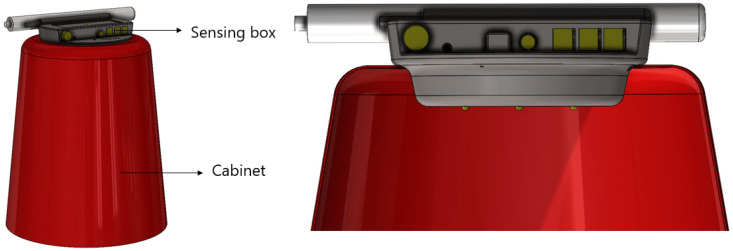
Section view cabinet and sensing box.

To allow ease production, the protective casing was designed considering the standard 3D printer sizes, thus the maximum dimension of sensing box was set to be 215 mm × 215 mm × 200 mm. These dimensions still allow the sensing box protective casing to be compact and easy to transport. Figure 8 presents the final design with a detailed section view of the sensing box integrated into the cabinet. To allow for easy assembly of these two parts, the casing design considers an angular shape so the sensing box effortlessly slides through the cabinet’s top part, guaranteeing a firm fix and alignment.

In order to provide a protective casing that allows for an intuitive interaction with the user, all exterior components as well as areas which the user should or not interact/block are duly marked. Buttons, interfaces, sensors are all labelled, and interruption symbols and written information indicate where the user should not interact or place objects on top (i.e., to avoid blocking of the light sensor).

The design includes slots to allow proper accommodation for humidity and pH probes, so that they integrate the sensing box when not used or while the box is being transported. The positioning of the slots considered the available internal space in order to guarantee the positioning of all external and internal parts as a single element, providing robustness to the structure as well as good mechanical properties for shock absorption and intense outdoor use.

**Figure 9:**
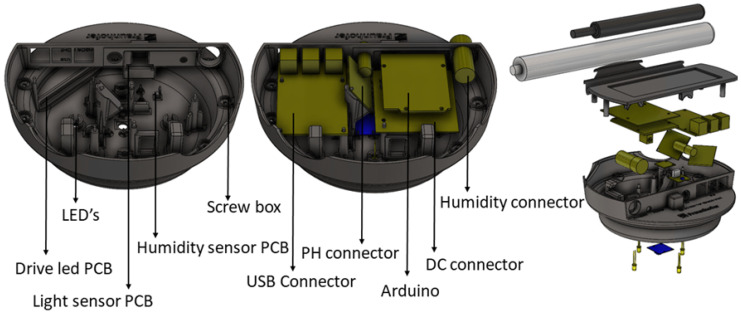
Sensing box–interior compartment.

With the external components duly identified, the casing design efforts shifted towards the interior of the sensing box. The main requirement here is that any user can build the sensing box following a DIY approach. For this reason, the interior of casing was designed to allow the hardware parts and cabling to be easily placed and avoid moving, as well as be sealed to avoid entrance of moisture and dust. Figure 9 details the placement of the main hardware parts of the sensing box.

By carefully considering the purpose of the IoT sensing platform, how it is assembled and transported, the environment it is expected to be used, and the different users that may handle it, the 3D-printed protective casing allows the components to be properly secured, resistance to harsh environmental condition, and robustness to drops (up to 1 meter), thus maximizing the duration of the sensing platform in field deployments.

Next, we present the different proofs of concept built for each of the components as well as specific validations carried out on the final prototype to help us having a single, integrated platform robust enough for the target application scenarios.

## 4. Proofs of Concept, Prototypes and Validations

This section is dedicated to the different proofs of concept built towards the integrated IoT sensing platform. It is worth noting that the initial proofs of concept have been built considering what the sensing platform should measure, that is, pH, moisture, texture, colour, air temperature, and light. Besides the Arduino and Raspberry Pi, we have used the Fraunhofer Portugal’s PANDLETS platform [54] and IoTiP sensor [55] technologies for the proofs of concept.

### 4.1. Proofs of Concept

Following the architecture presented in Figure 1, we created different prototypes related to the IoT sensing platform. These prototypes focused on evaluating each sensing element individually to assess their requirements and what would be the best way to integrate them in the sensing box. As our goal in this section is to solely show the evolution of the prototypes, we direct the reader for more details on the performed evaluation of this initial proofs of concept in [5].

#### 4.1.1. Soil Moisture

**Figure 10:**
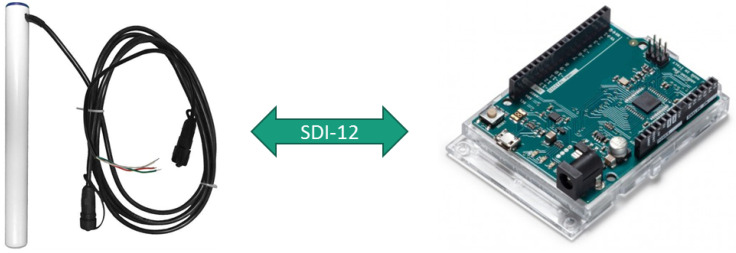
Prototype for soil moisture sensor.

The evaluation of the soil humidity sensor was performed using an Arduino and the Sentek drill & drop probe with three monitoring depths. As depicted in Figure 10 the interface between Arduino and Sentek probe is achieved by using the SDI-12 library from EnviroDIY [56].

With this prototype, it was possible to assess the requirements for the SDI-12 communication, namely its sampling requirements and message exchange protocol. Additionally, the prototype allowed us to validate an open source SDI-12 C-based library that can be directly used, or easily adapted, for use in the proposed IoT sensing platform, and its direct support to the Sentek drill & drop probe.

#### 4.1.2. Soil pH

**Figure 11:**
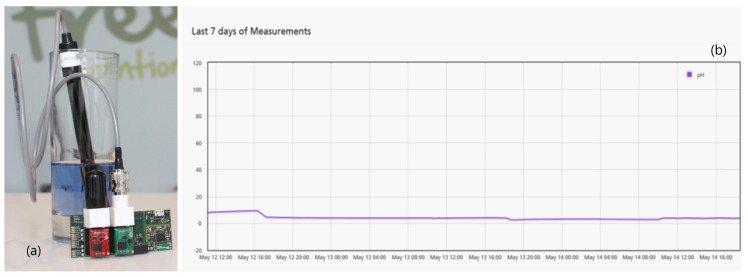
(**a**) Testing pH sensor; (**b**) PANDLETS web interface data graph.

To understand the requirements for the pH sensors we tested, as depicted in Figure 11a, an Atlas Scientific pH sensor with the Fraunhofer Portugal PANDLETS platform [54] (the first version of the IoTiP platform). The use of the PANDLETS platform instead of the IoTiP is related to the fact that the PANDLETS, with its Sensing+ model, already supported the communication with analogue-based sensors, while the IoTiP required the development of some hardware modules to support such feature.

With this test it was possible to assess the pH sensors requirements both in what regards the hardware interfaces needed to integrate with the analogue output returned by most sensors in the form of a BNC connector, and the firmware needed to interpret the received data. On this proof of concept, the data was being sent to a server inside Fraunhofer Portugal premises where it could be visualized in a web interface (cf., Figure 11b).

#### 4.1.3. Ambient Temperature

**Figure 12:**
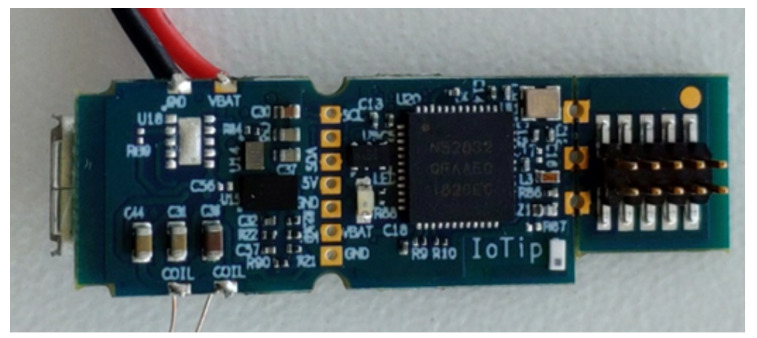
IoTiP PCB version.

As aforementioned, we also considered the IoTiP (cf., Figure 12) within the context of the IoT sensing platform given its modularity, radio interfaces and integrated light and temperature sensors.

In order to be fully compliant to the requirements of the IoT sensing platform, from the hardware standpoint IoTIP would require the addition of a PCB with Wi-Fi modules and different interfaces for the remaining sensors (e.g., pH, moisture, camera). Software-wise, IoTiP would need the implementation of the Wi-Fi and remaining sensor drivers. Additionally, the compliance with the low level of technical knowledge for further developments (following a DIY approach), and the availability of the components in the local market would not be fulfilled. Despite being focused on easing hardware and firmware development, the technical knowledge required for the development of new features on IoTiP is higher than with Arduino, since the IoTiP is not focused on DIY use cases. For these reasons, the IoTiP ended up not being used.

#### 4.1.4. Computer Vision

**Figure 13:**
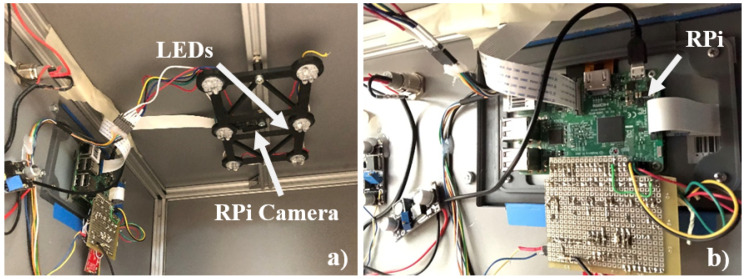
Computer vision prototype: (**a**) LEDs and RPi camera; (**b**) control unit including the Raspberry Pi.

The computer vision proof of concept was developed based on the Raspberry Pi and its camera. Figure 13 illustrates the initial setup of the computer vision component built for image acquisition and processing.

The main validation tasks considered in the proof of concept for the computer vision were:

- Testing a set of image acquisition equipment such as the one presented in Figure 13. The controlled image acquisition setup tries to avoid light leakage and improve image quality of the soil images acquired.
- Evaluating contrast enhancement and thresholding techniques.
- Testing feature extraction in different colour spaces - the images accessed in the RGB colour space can be transformed in different colour spaces, and some computations performed, including the statistical, frequency and spatial frequency.
- These were initially considered using OpenCV, but later dropped due to the machine learning approach adopted.

### 4.2. Prototype Validations

The assembly tests confirm that the camera can be tightly fitted in the slot, but some minor adjustments can be done, mostly tilt and pan, after all the electronics are in place and the images can be previewed. No problems were found on connecting the camera through CSI. The Raspberry Pi 4 must be rotated so that the USB-C power-in port can be exposed.

**Figure 14:**
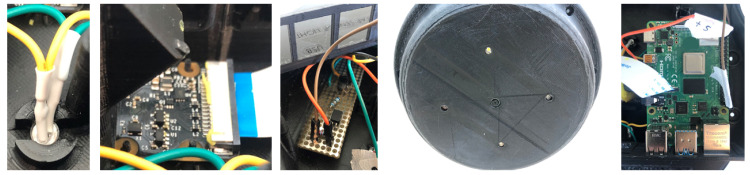
Assembly first component group of sensing box (from left to right): LED installation, camera, LED driver, bottom with camera, LED and USB Plug.

The connectivity between Raspberry Pi and Arduino is done through one of the USB-A ports. Concerning the power supply and control of the LED Driver through the GPIO interface, the Raspberry Pi kernel already comes with a GPIO driver that allows user-mode applications to control the GPIO pins (and LEDs connected to them) directly. However, we decided not to use it but to build a LED driver from scratch avoiding future redesigns, for cases when the current supply from the GPIO driver is not enough for the number of LEDs connected. This way the GPIO only signals the activation of the designed LED driver. The LED driver handles the current needed for the LEDs from the external energy source. All of these aspects can be observed in Figure 14. It is worth noting that the assembly process is divided into two main steps (as described next), thus making it easier to identify the components and avoid damaging them during assembly. In this way, it is possible to validate each step and evaluate the correctness of the process.

The first steps towards the final prototype consisted in the assembly of the first group of components into the sensing box (cf., Figure 14), namely camera, LEDs, Led Driver, and USB Plug. The LEDs are fixed through clips designed and integrated in the sensing box as well as maintaining both perfect alignment and verticality. The camera is aligned and leveled with the base of the sensing box fulfilling the horizontal relationship with the set of LEDs. The camera is secured and supported by four mounting boxes that use the holes in the camera’s PCB. The connection of the camera is made through flex cable and the LEDs with electric wire both with space and without interference with the other elements. The LED driver is also located at the base of the sensing box, secured in a dedicated slot and with space for the electrical wires.

**Figure 15:**
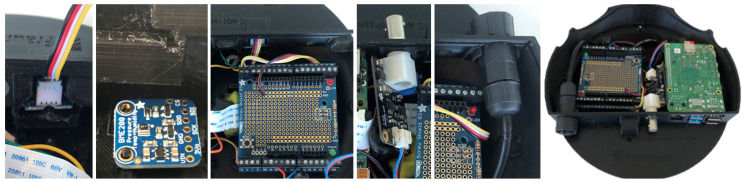
Assembly the top of the sensing box (from left to right): light and humidity sensor, DC plug and connector to the closed and fully assembled prototype.

After assembling the base of the sensing box, we followed with the assembling the second group of components. Figure 15 shows each element mounted, namely light sensor, humidity sensor, Arduino, DC plug, and humidity and PH connectors. The assembly is done in a way so each element uses its support, does not to interfere with the cable routing, and there is space for the individual assembly of each element and its replacement if necessary. The light sensor is inserted in a holder that works as a drawer for the sensor to slide and be centered with the hole to properly capture the outside light. The sensor is fixed in way that the connector and cables have enough space to be routed. The humidity sensor is located at the base of the sensing box, placed on the mounting box, and it also provides space for cable routing.

Arduino and DC are placed on their supports designed on the structure and then the connectors are glued. The pH PCB connector is placed on its supports and moisture sensor is placed in its specific location. With these two steps, the sensing box is assembled. The box is closed with the cover being screwed into place. This way, we have a robust exterior structure and an isolated interior.

**Figure 16:**
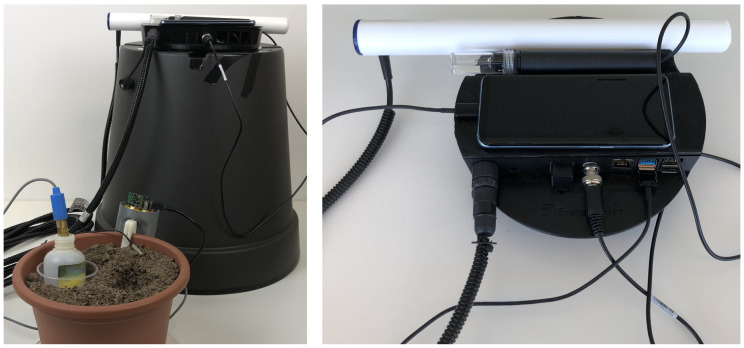
IoT sensing platform fully assembled prototype.

The final prototype with the sensing box and cabinet fully assembled are illustrated in Figure 16 and showing how the pH and moisture sensors are placed in the platform (left). Regarding shock resistance, the prototype has been validated and tested for 1-meter drops. Upon this, we can safely say that we managed to build a prototype that resorts to 3D printing technology and other tools that can be easily put together.

With the final prototype, we validated the interaction between the sensing box and the cabinet focusing on a solution that provides an easy connection, is able to create a functional work distance and is built into an unique structure with very good insulation from external factors (e.g., moisture, dust, light).

Regarding the soil classifier required by computer vision component, we validated considering the following steps: (i) preparation of a Python 3 environment; (ii) installation of tensorflow and keras framework with the specified version for the target device; (iii) management of the installation of all the other dependencies and libraries that keras and tensorflow frameworks rely on; (iv) deployment of the pre-trained and best soil image classification model with the respective weights; (v) adaptation of the main python script to load the best model and perform classification on every image taken.

The performance increase available in the Raspberry Pi 4 (comparing it with its predecessors) makes it a very competitive platform for machine learning at the edge. For this reason and due to its small power consumption, we selected this device to implement a pre-trained model to classify soil types in WRB classes. Also, it is possible to use a complete set of linux commands in the preparation of the running environment.

The validation of the communication between the Raspberry Pi and Arduino, as well as the sensor integration in the Arduino was performed along with the implementation of the sensing box software modules and communication sockets.

As for the validation of communication between the Raspberry Pi and Arduino, configuration messages were used for each sensor, and published in the ZeroMQ socket. The Arduino Readings SW Module correctly interpreted these messages and relayed them to the Serial Socket. The Arduino, on the other hand, was able to interpret the received instructions and communicate with the attached sensors. The collected readings were sent to the Arduino Readings SW Module, that relayed them to the ZeroMQ socket.

**Figure 17:**
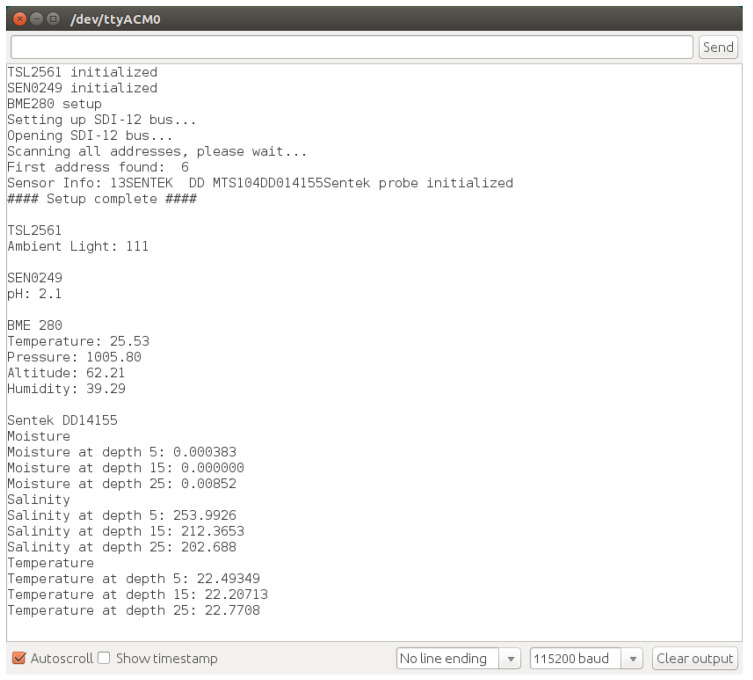
Sensor initialization and readings available at the Sensor Data Socket.

Figure 17 shows the terminal with the initialization and setup of the sensors (presented in Section 3.2.2), followed by the readings that are made available in the data socket, namely the ambient light (Seeed Studio TSL2561), pH (DFRobot SEN0249), temperature (Adafruit 2652 BME280), and moisture (Sentek drill & drop soil).

It is worth noting that both the Adafruit BME280 and Sentek drill & drop soil, provide more information than what is required from the IoT sensing platform, that is, temperature and moisture, respectively. However, this extra information (barometric pressure, altitude, air humidity, salinity and soil temperature) could be used according to the requirements of the application scenario (e.g., considering the specificity of the crop type).

These readings could then be consumed by any application subscribing the Sensor Data socket. For the communication between the Arduino and the sensors, we used the Arduino sensor drivers provided by the manufacturers, and the driver implemented in the proof of concept validation.

## 5. Conclusions and Future Work

This article proposed and validated an Internet of Things (IoT) sensing platform that provides information on the state of the soil and surrounding environment coupled with computer vision to classify the type of soil. The platform is based on low-cost, off-the-shelf hardware and software modularity, following a DIY approach and supporting further extension. The hardware is housed in a specifically designed robust 3D-printed casing to allow easy assembly, transport, and protection from the deployment environment.

The validation of the sensing box, the computer vision, and the casing was performed in a controlled, in-lab environment, to further fine tune and improve each component prior to testing and deployment in real work environment in Uganda and South Africa.

The SW architecture was tested and the socket communication validated, demonstrating that the sensing box is capable of receiving instructions (in the form of configuration messages) from an external device through the ZeroMQ configuration socket, interpret such instructions, collect sensor values via Arduino and communicate the results back to the external device via the ZeroMQ data socket.

The computer vision component was also validated concerning image acquisition and processing processes and quality of the captured image. The computer vision component is also capable of delivering processed information to the interface with the ZeroMQ sockets.

The 3D-printed protective casing was tested taking into consideration (i) how seamless the process of assembly is; (ii) the fitting and integration of all external and internal components; (iii) the fitting of the sensing box into the cabinet; (iv) the interaction of the user with the platform as whole; and (v) 1-meter drop shock resistance.

These in-lab tests carried out with IoT sensing platform have been satisfactory. With a simple setup, we have managed to get readings from the sensors available at data socket. Images were taken from the soil, and the computer vision algorithm is able to return the string value corresponding to the soil texture. These simple, and yet practical, tests demonstrate the viability of the IoT sensing platform to be employed as a support to digital farming.

Regarding future steps, which consider validation in the deployments in Uganda and South Africa, we have planned the following—stress tests shall be performed on the sensing box after this integration to understand how the solution behave in extreme scenario such as extended monitoring periods, high sampling frequencies, sensor or software communication errors, and so forth. On top of this, system validation in the field would allow to understand if the system can be mounted by users with low technical knowledge, and whether the DIY approach, in which the sensing box was constructed, requires improvements.

## Figures and Tables

**Table 1:** Results test set.

	3 Classes		7 Classes	
**Architectures**	**Accuracy**	**Loss**	**Accuracy**	**Loss**
VGG16	41.48%	8.2	24.66%	10.11
InceptionV3	36.73%	7.76	27.17%	5.07
MobileNetV2	38.07%	4.29	26.43%	5.2
NASNetMobile	38.74%	7.84	28.25%	5.04
ColorTexture	39.75%	1.96	23.81%	2.77

**Table 2:** Results validation set.

	3 Classes		7 Classes	
**Architectures**	**Accuracy**	**Loss**	**Accuracy**	**Loss**
VGG16	64.25%	0.92	26.88%	2.17
InceptionV3	57.21%	0.90	27.55%	5.13
MobileNetV2	56.87%	1.31	27.23%	5.17
NASNetMobile	61.07%	2.24	28.48%	1.85
ColorTexture	63.27%	0.97	32.45%	2.27
